# Tetra­imidazole­bis(trichloro­acetato)copper(II)

**DOI:** 10.1107/S1600536810017459

**Published:** 2010-05-22

**Authors:** Li-Min Li, Huan-Mei Guo, Fang-Fang Jian, Zeng-Hui Zhang, Ning Zhang

**Affiliations:** a Microscale Science Institute, Department of Chemistry and Chemical Engineering, Weifang University, Weifang 261061, People’s Republic of China; b Microscale Science Institute, Weifang University, Weifang 261061, People’s Republic of China; c Department of Chemistry and Chemical Engineering, Weifang University, Weifang 261061, People’s Republic of China

## Abstract

The title compound, [Cu(C_2_Cl_3_O_2_)_2_(C_3_H_4_N_2_)_4_], was prepared by the reaction of imidazole and trichloro­acetatocopper(II). The Cu^II^ atom adopts a distorted octa­hedral coordination geometry, binding the N atoms of four imidazole ligands and the carboxyl­ate O atoms of two trichloro­acetate anions. The mol­ecular structure and packing are stabilized by N—H⋯O hydrogen-bonding inter­actions. Close inter­molecular Cl⋯Cl contacts [3.498 (3) Å] are also found in the structure.

## Related literature

For background to work on metal-organic frameworks, see: Chen *et al.* (2001 ▶); Fang *et al.* (2005 ▶). For a related structure, see: Moncol *et al.* (2007 ▶).
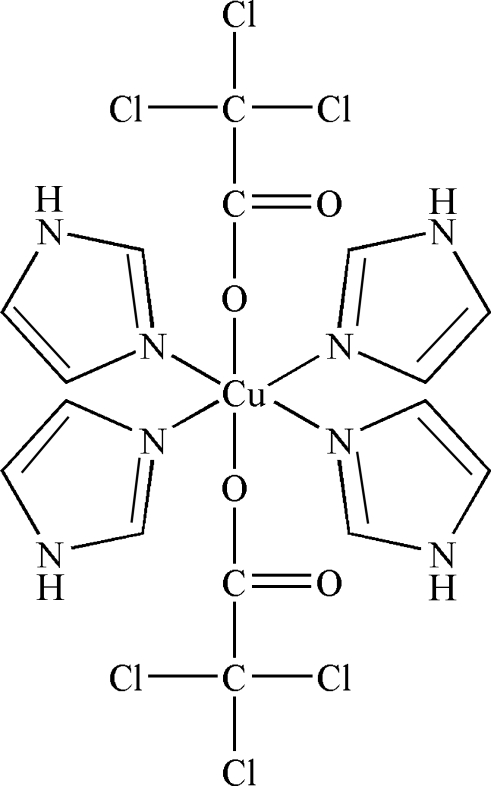


## Experimental

### 

#### Crystal data

[Cu(C_2_Cl_3_O_2_)_2_(C_3_H_4_N_2_)_4_]
*M* *_r_* = 660.61
Triclinic, 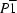

*a* = 10.054 (2) Å
*b* = 10.539 (2) Å
*c* = 12.959 (3) Å
α = 108.12 (3)°
β = 92.93 (3)°
γ = 95.18 (3)°
*V* = 1295.2 (4) Å^3^
*Z* = 2
Mo *K*α radiation
μ = 1.50 mm^−1^
*T* = 293 K
0.22 × 0.20 × 0.18 mm

#### Data collection

Bruker SMART CCD area-detector diffractometer
12377 measured reflections
5823 independent reflections
5048 reflections with *I* > 2σ(*I*)
*R*_int_ = 0.053

#### Refinement

*R*[*F* ^2^ > 2σ(*F* ^2^)] = 0.055
*wR*(*F* ^2^) = 0.168
*S* = 1.05
5823 reflections
316 parameters
H-atom parameters constrained
Δρ_max_ = 1.43 e Å^−3^
Δρ_min_ = −0.86 e Å^−3^

### 

Data collection: *SMART* (Bruker, 1997 ▶); cell refinement: *SAINT* (Bruker, 1997 ▶); data reduction: *SAINT*; program(s) used to solve structure: *SHELXS97* (Sheldrick, 2008 ▶); program(s) used to refine structure: *SHELXL97* (Sheldrick, 2008 ▶); molecular graphics: *SHELXTL* (Sheldrick, 2008 ▶); software used to prepare material for publication: *SHELXTL*.

## Supplementary Material

Crystal structure: contains datablocks global, I. DOI: 10.1107/S1600536810017459/sj2779sup1.cif

Structure factors: contains datablocks I. DOI: 10.1107/S1600536810017459/sj2779Isup2.hkl

Additional supplementary materials:  crystallographic information; 3D view; checkCIF report

## Figures and Tables

**Table 1:** Selected bond lengths (Å)

Cu1—N7	1.997 (2)
Cu1—N3	2.001 (2)
Cu1—N1	2.011 (3)
Cu1—N5	2.022 (3)
Cu1—O3	2.479 (2)
Cu1—O2	2.618 (2)

**Table 2:** Hydrogen-bond geometry (Å, °)

*D*—H⋯*A*	*D*—H	H⋯*A*	*D*⋯*A*	*D*—H⋯*A*
N8—H8*A*⋯O1^i^	0.86	2.03	2.885 (3)	177
N6—H6*A*⋯O4^ii^	0.86	2.02	2.854 (3)	164
N2—H2*A*⋯O1^iii^	0.86	1.96	2.790 (3)	162
N4—H4*B*⋯O4^iv^	0.86	1.93	2.764 (3)	162

Symmetry codes: (i) 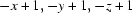; (ii) 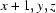; (iii) 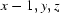; (iv) 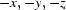.

**Fig. 1.**
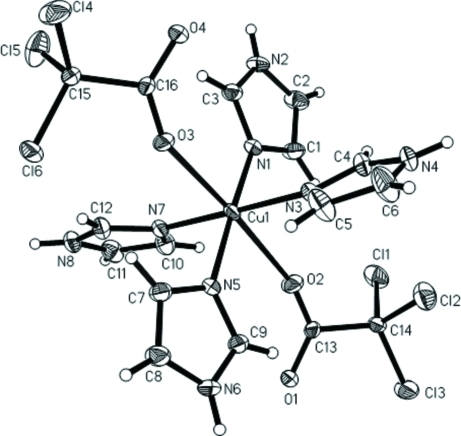
The structure of the title compound showing 30% probability displacement ellipsoids and the atom-numbering scheme.

**Fig. 2.**
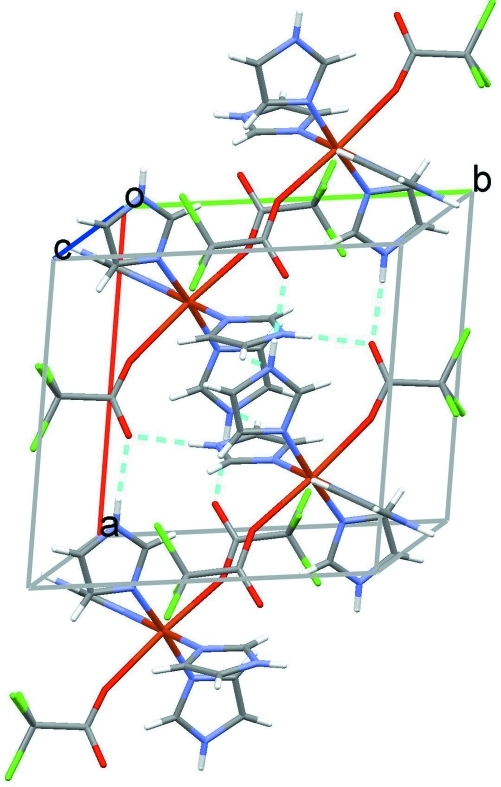
Crystal packing of the title compound viewed down the c axis.

## supplementary crystallographic information

### Comment

Metal-organic framework coordination polymers have attracted tremendous attention because of their molecular topologies and their potentially useful ion exchange, adsorption, catalytic and magnetic properties (Chen *et al.*, 2001; Fang *et al.*, 2005 ). In order to search for new complexes of this type, we synthesized the title compound and report its crystal structure here.

The title structure contains one copper(II) cation, four imidazole ligands and two trichloroacetate anions. The coordination sphere of the copper(II) ion is best described as a slightly distorted octahedron. The Cu—N bond lengths are in agreement with those reported recently (Moncol *et al.*, 2007). The crystal packing is stabilized by C—H···O and N—H···O hydrogen interaction (Table 1).

### Experimental

The title compound was obtained by adding imidazole(4 mmol) dropwise to a solution of copper(II) trichloroacetate acid (1 mmol) in ethanol (30 ml) with stirring for 1 hour at room temperature. A blue solution formed and after a few days rod-like crystals precipitated.

### Refinement

All H-atoms were positioned geometrically and refined using a riding model with d(C-H) = 0.93Å,  U_iso_=1.2U_eq_ (C) for aromatic H atoms and 0.86Å, U_iso_ = 1.2U_eq_ (N) for the NH groups.

### Crystal data

**Table:**

[Cu(C_2_Cl_3_O_2_)_2_(C_3_H_4_N_2_)_4_]	*Z* = 2
*M**_r_* = 660.61	*F*(000) = 662
Triclinic, *P*1	*D*_x_ = 1.694 Mg m^−^^3^
Hall symbol: -P 1	Mo *K*α radiation, λ = 0.71073 Å
*a* = 10.054 (2) Å	Cell parameters from 2238 reflections
*b* = 10.539 (2) Å	θ = 2.1–26.3°
*c* = 12.959 (3) Å	µ = 1.50 mm^−^^1^
α = 108.12 (3)°	*T* = 293 K
β = 92.93 (3)°	Rod, blue
γ = 95.18 (3)°	0.22 × 0.20 × 0.18 mm
*V* = 1295.2 (4) Å^3^	

### Data collection

**Table:**

Bruker SMART CCD area-detector diffractometer	5048 reflections with *I* > 2σ(*I*)
Radiation source: fine-focus sealed tube	*R*_int_ = 0.053
graphite	θ_max_ = 27.5°, θ_min_ = 3.0°
φ and ω scans	*h* = −13→10
12377 measured reflections	*k* = −13→13
5823 independent reflections	*l* = −16→16

### Refinement

**Table:**

Refinement on *F*^2^	Primary atom site location: structure-invariant direct methods
Least-squares matrix: full	Secondary atom site location: difference Fourier map
*R*[*F*^2^ > 2σ(*F*^2^)] = 0.055	Hydrogen site location: inferred from neighbouring sites
*wR*(*F*^2^) = 0.168	H-atom parameters constrained
*S* = 1.05	*w* = 1/[σ^2^(*F*_o_^2^) + (0.1013*P*)^2^ + 0.8989*P*] where *P* = (*F*_o_^2^ + 2*F*_c_^2^)/3
5823 reflections	(Δ/σ)_max_ < 0.001
316 parameters	Δρ_max_ = 1.43 e Å^−^^3^
0 restraints	Δρ_min_ = −0.85 e Å^−^^3^

### Special details

**Table:**

Geometry. All esds (except the esd in the dihedral angle between two l.s. planes) are estimated using the full covariance matrix. The cell esds are taken into account individually in the estimation of esds in distances, angles and torsion angles; correlations between esds in cell parameters are only used when they are defined by crystal symmetry. An approximate (isotropic) treatment of cell esds is used for estimating esds involving l.s. planes.
Refinement. Refinement of *F*^2^ against ALL reflections. The weighted *R*-factor wR and goodness of fit *S* are based on *F*^2^, conventional *R*-factors *R* are based on *F*, with *F* set to zero for negative *F*^2^. The threshold expression of *F*^2^ > σ(*F*^2^) is used only for calculating *R*-factors(gt) etc. and is not relevant to the choice of reflections for refinement. *R*-factors based on *F*^2^ are statistically about twice as large as those based on *F*, and *R*- factors based on ALL data will be even larger.

### Fractional atomic coordinates and isotropic or equivalent isotropic displacement parameters (Å^2^)

**Table:**

	*x*	*y*	*z*	*U*_iso_*/*U*_eq_	
Cu1	0.26177 (3)	0.25376 (3)	0.24476 (3)	0.02655 (14)	
Cl1	0.41741 (11)	−0.13876 (10)	0.38566 (9)	0.0542 (3)	
Cl4	−0.12008 (10)	0.61045 (11)	0.09196 (10)	0.0593 (3)	
Cl6	0.14287 (9)	0.67968 (9)	0.19926 (13)	0.0687 (4)	
Cl2	0.44972 (12)	−0.16467 (11)	0.16082 (9)	0.0619 (3)	
Cl3	0.67814 (10)	−0.15040 (9)	0.30617 (13)	0.0744 (4)	
Cl5	−0.08098 (15)	0.63279 (11)	0.31833 (10)	0.0716 (4)	
N7	0.2999 (2)	0.3951 (2)	0.3900 (2)	0.0290 (5)	
N3	0.2209 (2)	0.1099 (2)	0.1006 (2)	0.0296 (5)	
N1	0.1157 (2)	0.1667 (2)	0.3089 (2)	0.0294 (5)	
O1	0.6441 (2)	0.1316 (2)	0.3576 (2)	0.0412 (6)	
O3	0.1177 (2)	0.4023 (2)	0.1870 (2)	0.0390 (5)	
C16	0.0052 (3)	0.4292 (3)	0.1668 (2)	0.0271 (6)	
N5	0.4180 (2)	0.3358 (2)	0.1859 (2)	0.0269 (5)	
C14	0.5229 (3)	−0.0894 (3)	0.2966 (3)	0.0349 (7)	
N8	0.3302 (3)	0.5850 (3)	0.5243 (2)	0.0424 (7)	
H8A	0.3349	0.6695	0.5591	0.051*	
C15	−0.0107 (3)	0.5819 (3)	0.1915 (3)	0.0357 (7)	
C4	0.1621 (3)	−0.0146 (3)	0.0788 (3)	0.0377 (7)	
H4A	0.1307	−0.0507	0.1311	0.045*	
C3	−0.0074 (3)	0.1991 (3)	0.3191 (3)	0.0335 (6)	
H3A	−0.0390	0.2708	0.3018	0.040*	
N6	0.6185 (2)	0.3773 (3)	0.1383 (2)	0.0337 (6)	
H6A	0.7006	0.3686	0.1245	0.040*	
O2	0.4247 (2)	0.1096 (2)	0.3115 (2)	0.0460 (6)	
C12	0.2979 (4)	0.5269 (3)	0.4173 (3)	0.0394 (7)	
H12A	0.2769	0.5727	0.3689	0.047*	
N2	−0.0805 (3)	0.1150 (3)	0.3579 (2)	0.0376 (6)	
H2A	−0.1633	0.1184	0.3713	0.045*	
C13	0.5316 (3)	0.0686 (3)	0.3258 (3)	0.0294 (6)	
C9	0.5368 (3)	0.2938 (3)	0.1716 (3)	0.0313 (6)	
H9A	0.5608	0.2159	0.1833	0.038*	
C1	0.1205 (3)	0.0559 (3)	0.3430 (3)	0.0399 (7)	
H1A	0.1957	0.0112	0.3454	0.048*	
N4	0.1536 (3)	−0.0808 (3)	−0.0272 (3)	0.0433 (7)	
H4B	0.1195	−0.1626	−0.0581	0.052*	
C8	0.5480 (3)	0.4792 (3)	0.1302 (3)	0.0424 (8)	
H8B	0.5789	0.5522	0.1086	0.051*	
C2	−0.0014 (3)	0.0224 (4)	0.3726 (3)	0.0438 (8)	
H2B	−0.0262	−0.0491	0.3978	0.053*	
C7	0.4245 (3)	0.4526 (3)	0.1596 (3)	0.0381 (7)	
H7A	0.3546	0.5052	0.1618	0.046*	
C11	0.3542 (4)	0.4870 (4)	0.5681 (3)	0.0456 (8)	
H11A	0.3785	0.4980	0.6409	0.055*	
C6	0.2081 (5)	0.0029 (5)	−0.0779 (3)	0.0653 (13)	
H6B	0.2160	−0.0161	−0.1523	0.078*	
C5	0.2490 (5)	0.1207 (4)	0.0017 (3)	0.0543 (10)	
H5A	0.2902	0.1974	−0.0095	0.065*	
C10	0.3354 (4)	0.3696 (3)	0.4845 (3)	0.0405 (7)	
H10A	0.3452	0.2852	0.4904	0.049*	
O4	−0.1012 (2)	0.3534 (2)	0.1345 (2)	0.0390 (6)	

### Atomic displacement parameters (Å^2^)

**Table:**

	*U*^11^	*U*^22^	*U*^33^	*U*^12^	*U*^13^	*U*^23^
Cu1	0.0222 (2)	0.0225 (2)	0.0301 (2)	−0.00600 (13)	0.00312 (13)	0.00353 (15)
Cl1	0.0542 (6)	0.0461 (5)	0.0682 (6)	−0.0106 (4)	0.0040 (4)	0.0314 (4)
Cl4	0.0390 (5)	0.0648 (6)	0.0921 (8)	0.0136 (4)	−0.0025 (5)	0.0498 (6)
Cl6	0.0311 (5)	0.0322 (5)	0.1425 (12)	−0.0079 (3)	−0.0069 (5)	0.0329 (5)
Cl2	0.0673 (7)	0.0517 (6)	0.0537 (6)	−0.0014 (5)	0.0007 (5)	0.0010 (4)
Cl3	0.0299 (5)	0.0347 (5)	0.1577 (13)	0.0097 (4)	−0.0054 (6)	0.0295 (6)
Cl5	0.0955 (9)	0.0523 (6)	0.0649 (7)	0.0291 (6)	0.0273 (6)	0.0068 (5)
N7	0.0274 (12)	0.0239 (12)	0.0307 (12)	−0.0021 (9)	0.0016 (9)	0.0032 (9)
N3	0.0265 (12)	0.0227 (12)	0.0352 (13)	−0.0011 (9)	0.0000 (9)	0.0045 (9)
N1	0.0220 (12)	0.0265 (12)	0.0366 (13)	−0.0031 (9)	0.0016 (9)	0.0072 (10)
O1	0.0255 (11)	0.0243 (11)	0.0680 (16)	−0.0004 (8)	0.0045 (10)	0.0072 (10)
O3	0.0243 (11)	0.0312 (11)	0.0648 (16)	0.0079 (9)	−0.0020 (10)	0.0193 (10)
C16	0.0235 (13)	0.0207 (13)	0.0377 (15)	0.0043 (10)	0.0042 (10)	0.0096 (10)
N5	0.0215 (11)	0.0241 (11)	0.0319 (12)	−0.0026 (9)	0.0001 (9)	0.0059 (9)
C14	0.0225 (14)	0.0250 (14)	0.056 (2)	0.0005 (11)	−0.0017 (12)	0.0128 (13)
N8	0.0403 (16)	0.0304 (14)	0.0435 (16)	−0.0018 (11)	0.0021 (12)	−0.0050 (12)
C15	0.0238 (14)	0.0259 (14)	0.058 (2)	0.0039 (11)	0.0032 (13)	0.0142 (13)
C4	0.0408 (18)	0.0237 (14)	0.0433 (18)	−0.0025 (12)	0.0015 (13)	0.0049 (12)
C3	0.0240 (14)	0.0353 (16)	0.0424 (17)	0.0007 (11)	0.0042 (11)	0.0144 (13)
N6	0.0193 (12)	0.0384 (14)	0.0436 (15)	0.0014 (10)	0.0066 (10)	0.0132 (11)
O2	0.0285 (12)	0.0370 (13)	0.0778 (19)	0.0111 (10)	0.0030 (11)	0.0239 (12)
C12	0.0413 (18)	0.0271 (15)	0.0458 (19)	0.0002 (13)	−0.0012 (14)	0.0075 (13)
N2	0.0210 (12)	0.0474 (16)	0.0459 (15)	−0.0026 (11)	0.0043 (10)	0.0184 (12)
C13	0.0260 (14)	0.0209 (13)	0.0437 (16)	0.0048 (10)	0.0085 (11)	0.0121 (11)
C9	0.0257 (14)	0.0303 (14)	0.0398 (16)	0.0050 (11)	0.0042 (11)	0.0132 (12)
C1	0.0252 (15)	0.0426 (18)	0.059 (2)	0.0046 (13)	0.0035 (13)	0.0259 (15)
N4	0.0420 (16)	0.0270 (14)	0.0465 (17)	−0.0031 (11)	−0.0039 (12)	−0.0062 (12)
C8	0.0301 (16)	0.0394 (18)	0.067 (2)	0.0030 (13)	0.0102 (15)	0.0290 (16)
C2	0.0341 (18)	0.049 (2)	0.056 (2)	−0.0062 (14)	0.0003 (14)	0.0309 (17)
C7	0.0255 (15)	0.0341 (16)	0.062 (2)	0.0053 (12)	0.0068 (13)	0.0243 (15)
C11	0.049 (2)	0.048 (2)	0.0307 (16)	−0.0056 (16)	0.0007 (13)	0.0033 (14)
C6	0.087 (3)	0.059 (3)	0.0338 (19)	−0.020 (2)	0.0015 (19)	−0.0010 (18)
C5	0.073 (3)	0.047 (2)	0.0355 (18)	−0.0206 (19)	−0.0033 (17)	0.0105 (15)
C10	0.048 (2)	0.0372 (17)	0.0346 (16)	0.0008 (14)	0.0011 (13)	0.0101 (13)
O4	0.0229 (10)	0.0261 (11)	0.0637 (16)	−0.0024 (8)	−0.0011 (9)	0.0102 (10)

### Geometric parameters (Å, °)

**Table:**

Cu1—N7	1.997 (2)	N8—H8A	0.8600
Cu1—N3	2.001 (2)	C4—N4	1.327 (4)
Cu1—N1	2.011 (3)	C4—H4A	0.9300
Cu1—N5	2.022 (3)	C3—N2	1.333 (4)
Cu1—O3	2.479 (2)	C3—H3A	0.9300
Cu1—O2	2.618 (2)	N6—C9	1.333 (4)
Cl1—C14	1.768 (4)	N6—C8	1.367 (4)
Cl4—C15	1.767 (4)	N6—H6A	0.8600
Cl6—C15	1.757 (3)	O2—C13	1.220 (4)
Cl2—C14	1.778 (4)	C12—H12A	0.9300
Cl3—C14	1.754 (3)	N2—C2	1.364 (5)
Cl5—C15	1.768 (4)	N2—H2A	0.8600
N7—C12	1.325 (4)	C9—H9A	0.9300
N7—C10	1.370 (4)	C1—C2	1.353 (5)
N3—C4	1.328 (4)	C1—H1A	0.9300
N3—C5	1.362 (5)	N4—C6	1.350 (6)
N1—C3	1.316 (4)	N4—H4B	0.8600
N1—C1	1.375 (4)	C8—C7	1.348 (5)
O1—C13	1.240 (4)	C8—H8B	0.9300
O3—C16	1.226 (4)	C2—H2B	0.9300
C16—O4	1.245 (4)	C7—H7A	0.9300
C16—C15	1.565 (4)	C11—C10	1.358 (5)
N5—C9	1.312 (4)	C11—H11A	0.9300
N5—C7	1.372 (4)	C6—C5	1.357 (5)
C14—C13	1.582 (4)	C6—H6B	0.9300
N8—C12	1.339 (5)	C5—H5A	0.9300
N8—C11	1.358 (5)	C10—H10A	0.9300
			
N7—Cu1—N3	178.78 (10)	N3—C4—H4A	124.3
N7—Cu1—N1	87.99 (10)	N1—C3—N2	110.6 (3)
N3—Cu1—N1	90.80 (11)	N1—C3—H3A	124.7
N7—Cu1—N5	91.08 (10)	N2—C3—H3A	124.7
N3—Cu1—N5	90.11 (10)	C9—N6—C8	107.4 (3)
N1—Cu1—N5	175.95 (10)	C9—N6—H6A	126.3
N7—Cu1—O3	88.90 (10)	C8—N6—H6A	126.3
N3—Cu1—O3	91.37 (9)	N7—C12—N8	110.6 (3)
N1—Cu1—O3	95.17 (9)	N7—C12—H12A	124.7
N5—Cu1—O3	88.70 (9)	N8—C12—H12A	124.7
N7—Cu1—O2	88.42 (10)	C3—N2—C2	108.2 (3)
N3—Cu1—O2	91.36 (10)	C3—N2—H2A	125.9
N1—Cu1—O2	87.03 (9)	C2—N2—H2A	125.9
N5—Cu1—O2	89.06 (9)	O2—C13—O1	129.8 (3)
O3—Cu1—O2	176.47 (7)	O2—C13—C14	113.8 (3)
C12—N7—C10	105.8 (3)	O1—C13—C14	116.3 (2)
C12—N7—Cu1	130.0 (2)	N5—C9—N6	111.3 (3)
C10—N7—Cu1	124.2 (2)	N5—C9—H9A	124.4
C4—N3—C5	104.8 (3)	N6—C9—H9A	124.4
C4—N3—Cu1	129.0 (2)	C2—C1—N1	109.2 (3)
C5—N3—Cu1	126.2 (2)	C2—C1—H1A	125.4
C3—N1—C1	106.1 (3)	N1—C1—H1A	125.4
C3—N1—Cu1	126.4 (2)	C4—N4—C6	107.9 (3)
C1—N1—Cu1	127.3 (2)	C4—N4—H4B	126.0
O3—C16—O4	129.6 (3)	C6—N4—H4B	126.0
O3—C16—C15	116.0 (3)	C7—C8—N6	106.1 (3)
O4—C16—C15	114.3 (2)	C7—C8—H8B	126.9
C9—N5—C7	105.8 (3)	N6—C8—H8B	126.9
C9—N5—Cu1	127.4 (2)	C1—C2—N2	105.9 (3)
C7—N5—Cu1	126.8 (2)	C1—C2—H2B	127.0
C13—C14—Cl3	114.2 (2)	N2—C2—H2B	127.0
C13—C14—Cl1	108.4 (2)	C8—C7—N5	109.4 (3)
Cl3—C14—Cl1	108.81 (19)	C8—C7—H7A	125.3
C13—C14—Cl2	108.8 (2)	N5—C7—H7A	125.3
Cl3—C14—Cl2	107.99 (19)	C10—C11—N8	106.2 (3)
Cl1—C14—Cl2	108.56 (17)	C10—C11—H11A	126.9
C12—N8—C11	108.0 (3)	N8—C11—H11A	126.9
C12—N8—H8A	126.0	N4—C6—C5	105.9 (4)
C11—N8—H8A	126.0	N4—C6—H6B	127.0
C16—C15—Cl6	112.6 (2)	C5—C6—H6B	127.0
C16—C15—Cl4	111.9 (2)	C6—C5—N3	110.0 (4)
Cl6—C15—Cl4	107.7 (2)	C6—C5—H5A	125.0
C16—C15—Cl5	106.0 (2)	N3—C5—H5A	125.0
Cl6—C15—Cl5	109.80 (19)	C11—C10—N7	109.4 (3)
Cl4—C15—Cl5	108.70 (17)	C11—C10—H10A	125.3
N4—C4—N3	111.3 (3)	N7—C10—H10A	125.3
N4—C4—H4A	124.3		

### Hydrogen-bond geometry (Å, °)

**Table:**

*D*—H···*A*	*D*—H	H···*A*	*D*···*A*	*D*—H···*A*
N8—H8A···O1^i^	0.86	2.03	2.885 (3)	177
N6—H6A···O4^ii^	0.86	2.02	2.854 (3)	164
N2—H2A···O1^iii^	0.86	1.96	2.790 (3)	162
N4—H4B···O4^iv^	0.86	1.93	2.764 (3)	162

Symmetry codes: (i) −*x*+1, −*y*+1, −*z*+1; (ii) *x*+1, *y*, *z*; (iii) *x*−1, *y*, *z*; (iv) −*x*, −*y*, −*z*.
